# Rejection of Unfair Offers Can Be Driven by Negative Emotions, Evidence from Modified Ultimatum Games with Anonymity

**DOI:** 10.1371/journal.pone.0039619

**Published:** 2012-06-28

**Authors:** Ning Ma, Nan Li, Xiao-Song He, De-Lin Sun, Xiaochu Zhang, Da-Ren Zhang

**Affiliations:** CAS Key Laboratory of Brain Function and Disease and School of Life Sciences, University of Science and Technology of China, Hefei, Anhui, China; University of Bologna, Italy

## Abstract

The rejection of unfair offers can be affected by both negative emotions (e.g. anger and moral disgust) and deliberate cognitive processing of behavioral consequences (e.g. concerns of maintaining social fairness and protecting personal reputation). However, whether negative emotions are sufficient to motivate this behavior is still controversial. With modified ultimatum games, a recent study (Yamagishi T, *et al.* (2009) *Proc Natl Acad Sci USA* 106∶11520–11523) found that people reject unfair offers even when this behavior increases inequity, and even when they could not communicate to the proposers. Yamagishi suggested that rejection of unfair offers could occurr without people’s concerning of maintaining social fairness, and could be driven by negative emotions. However, as anonymity was not sufficiently guaranteed in Yamagishi’s study, the rejection rates in their experiments may have been influenced by people’s concerns of protecting personal reputation (reputational concerns) in addition to negative emotions; thus, it was unclear whether the rejection was driven by negative emotions, or by reputational concerns, or both. In the present study, with specific methods to ensure anonymity, the effect of reputational concerns was successfully ruled out. We found that in a private situation in which rejection could not be driven by reputational concerns, the rejection rates of unfair offers were significantly larger than zero, and in public situations in which rejection rates could be influenced by both negative emotions and reputational concerns, rejection rates were significantly higher than that in the private situation. These results, together with Yamagishi’s findings, provided more complete evidence suggesting (a) that the rejection of unfair offers can be driven by negative emotions and (b) that deliberate cognitive processing of the consequences of the behavior can increase the rejection rate, which may benefit social cooperation.

## Introduction

The rejection of unfair offers is crucial in maintaining social fairness and cooperation [1], [2]. Ultimatum game (UG) was often employed to characterize people’s attitude toward unfair offers [3]. In UG, one participant (the responder) has to decide to either accept or reject an offer of money made by another player (the proposer) whose task is to distribute a sum of money between two of them. If the offer is accepted, both receive the money, otherwise, no one receives anything. It has been found that the responder often rejects unfair offers even though he/she does not know who the proposer is and the game is played only once [2], [4]. Rejection of unfair offers in UG can be regarded as a kind of altruism which is important for the evolution of reciprocity and social cooperation [5].

Recently, it has been suggested that in the UG, (a) negative emotions play an important role in the responder’s rejection behaviors [4], [6], [7] and (b) deliberate cognitive processing of the consequences of the rejection behavior, such as concerns of maintaining social fairness and protecting personal reputation, also significantly influence responders’ decisions [8], [9], [10], [11]. Therefore, an interesting and important question arises. Is the cognitive processing of behavioral consequences necessary for rejection or are negative emotions sufficient to drive rejection behaviors? This question, however, is still controversial [12], [13].

A recent related behavioral study provided some evidence to address this question [14]. Yamagishi and his colleagues used two modified versions of the UG, “impunity game” (IG) and “private impunity game” (PIG). In both games, the proposer will receive the money regardless of the responder’s choice, whereas the responder can only receive the money by accepting the proposal. In IG, the proposer will be informed of the responder’s choice, while in PIG, the proposer will not be. In Yamagishi’s study, substantial rejection rates (i.e., significant >0) were found in IG and PIG, which demonstrated that responders reject unfair offers even when this behavior increases inequity. As the rejection of unfair offers that increases inequity cannot be explained by the responder’s deliberate cognitive processing of their behavioral consequences, such as concerns of maintaining social fairness, Yamagishi interpreted their findings by the model of emotion as a commitment device, which proposed that: negative emotional responses to unfair treatment, such as anger or moral disgust, lead people to reject unfair offers.

For the different settings in IG and PIG, Yamagishi proposed that, in IG, responders may imagine the proposers’ feelings of knowing the responders’ choices. Thus, the responders could conclude that the rejection behaviors, as a “symbolic punishment to the unfair proposer”, may be helpful in maintaining social fairness [14]. In other words, in contrast to PIG, the decision of rejection rates in IG may also be influenced by the responders’ deliberate cognitive processing of their behavioral consequences in addition to negative emotions. However, in their study, the rejection rates were not significantly different between IG and PIG, indicating that (by Yamagishi’s reasoning), in IG, the concerns of the consequences of the rejection behavior may not significantly influence the rejection rates. Therefore, Yamagishi suggested that the emotion “as a commitment device seem strong enough to dictate that one’s behavior be consistent regardless of the consequences of the behavior”. In other words, Yamagishi suggested that the rejection of unfair offers could be dominated by negative emotions in their situations.

However, we found that, in Yamagishi’s study, responders’ anonymity was not sufficiently guaranteed, i.e., when the responders were making decisions, they may have perceived that their individual choices were known by the experimenters ^1^. In social decision-making tasks, it has been found that the presence of an audience, even if it is only the experimenter, can cause significant changes in participants’ behaviors [15], and this effect can be explained as the participant’s concerns of protecting personal reputation (reputational concerns) during the task [16], [17]. Therefore, the experiment setting Yamagishi used may have led to rejections in both games being influenced by the responders’ reputational concerns in addition to negative emotions. People might reject unfair offers during IG and PIG based on the consideration of protecting personal reputation besides the negative emotions. Therefore, it is still unclear whether people’s rejection of unfair offers can be driven by negative emotions.

In our current study, using specific setting in which no one could know responders individual decisions, responders’ anonymity was ensured in our private situation, thus, in this situation, the effect of reputational concerns would be ruled out. Consequently, if responders rejected unfair offers in the private situation, they could be driven by negative emotions. We hypothesized that the rejection rates in the private situation would be lower than those in public situations. More importantly, by comparing the rejection rates in the private situation with zero, we may demonstrate whether the rejection was only based on the reputational concerns, thus find further evidence to address the question: can the rejection of unfair offers be driven by negative emotions?

In our study, we renamed the two games as “informed impunity game” (informed-IG, for Yamagishi’s IG) and “non-informed impunity game” (non-informed-IG, for PIG). We examined responders’ rejection rates in these two games when they made choices with anonymity or been observed by an experimenter. Participants’ rejection in the non-informed-IG in anonymous condition can be regarded as taking place in a private situation in which no one (including the experimenter) would know a participant’s individual decision, while other situations can be regarded as public situations.

**Note 1.** In Yamagishi’s study, in the “strategy method” and “one-shot method” settings, each participant received an envelope from an experimenter, which contained the decision sheets. The participant then made a decision individually and sealed it into the envelope without being seen by anyone else. After that, the participant gave the envelope to an experimenter and waited, and later on, the same experimenter would return the envelope back to the participant (sealed, possibly containing money, according to the decision made by the participant). Although participants were told that they would never directly interact with the experimenters who knew their individual choices, they could have felt that their choices were known by the experimenter who had taken and returned their envelopes. In the “repeated one-shot method,” participants’ choices were recorded individually with computers, thus participants may also have felt that the experimenters knew their individual choices. In other words, since it was possible for experimenters to know participants’ individual choices in Yamagishi’s study, it cannot be ruled out the possibility that participants sensed that their decisions were made in public situations. In our present study, specific settings in the private situation eliminated the possibility for anyone to be aware of each participant’s individual choice (see Materials and Methods). Consequently, participants could feel assured that their decisions were not known by anyone (including the experimenters).

## Materials and Methods

### Ethics Statement

The study and the consent procedure were approved by the Human Research Ethics Committee of the University of Science & Technology of China (USTC) according to the principles expressed in the Declaration of Helsinkioral.

As the key of this study was to obtain participants’ rejection rates on unfair offers under truly private situation, anonymity must be guaranteed in our study. Therefore, beside our specific experiment settings, to further ensure participants’ sense of anonymity, we obtained oral consents of participants without requiring them to give their personal identification, which made them feel less identifiable. In a previous study with anonymity, researchers also did not obtain information about participant’s personal identification for ensuring anonymity (Kurzban R, et. al. (2007) Evolution and Human Behavior 28: 75–84).

In our study, the oral consent was obtained in following procedure. After complete description of the study to the participants, participants who did not want to continue were allowed to leave, so that participant’s stay in the classroom was regarded as his/her consent of involvement in the study. This procedure was supervised by at least three experimenters and documented (written) by an experimenter.

Regarding the participants aged 16/17 - these young students were of comparable intelligence and ability to adult students in the USTC, and able to take charge of their behaviors including taking part in some simple behavior studies. According to the law in China: “A minor aged 10 or over shall be a person with limited capacity for civil conduct and may engage in civil activities appropriate to his age and intellect; in other civil activities, he shall be represented by his agent ad litem or participate with the consent of his agent ad litem”, from the “General Principles of the Civil Law of the People’s Republic of China”, Article 12 in Chapter II. Therefore, we got the consents from the participants’ selves rather than from their legal guardians (usually their parents) for the involvement of our current simple behavior study.

### Participants

Four hundred and twenty seven students (329 males and 98 females, aged 16 to 29) at the University of Science & Technology of China, Hefei, Anhui, China, participated in our experiment. All played the role of responder. We employed a two-by-two design, including two games, “informed impunity game” (informed-IG) and the “non-informed impunity game,” (non-informed-IG) in each of two conditions, anonymous and experimenter conditions. Both the game and condition factors were manipulated as between-participants factors. Each participant performed one game in one condition with two paradigms: a behavioral paradigm and a questionnaire paradigm.

### Experiment Settings and Procedure

In the present study, we adapted the settings that had been used by previous studies on other tasks [15], [18] with specific methods to guarantee anonymity. Our data were obtained in several testing sessions (see Text S1 for detailed information about each testing session). In each session, we first recruited 11 to 76 participants and required them to stay in a classroom. One experimenter then introduced the requirements of the experiment. For ensuring participants’ sense of anonymity, we obtained oral consents of participants in following procedure. After complete description of the study to the participants, participants who did not want to continue were allowed to leave, so that participant’s stay in the classroom was regarded as his/her consent of involvement in the study. This procedure was supervised by at least three experimenters and documented (written) by an experimenter. The study and the consent procedure were approved by the Human Research Ethics Committee of the University of Science & Technology of China.

One at a time, each participant then went to the front of the classroom and took an envelope from a box that contained about 200 envelopes of different types according to our task design. Each envelope contained (a) the rules, which were printed on a sheet of paper, and explained whether the participant was playing the informed IG or the non-informed IG; (b) the “real” proposal by a “proposer”: a number-stamp printed on the paper represented the money a proposer offered; no actual proposers were involved in any of our experiments, but participant (who played the role of responders) was told that he/she would receive a proposal from a “real” proposer who had “participated” an earlier session of our experiments and been required to “give” a proposal in the games; (c) the money offered to the responder according to the proposal; and (d) a decision sheet with all nine possible proposals from 9∶1 (proposer receives 9 Chinese Yuan, and responder receives 1 Chinese Yuan) to 1∶9 (proposer receives 1 Chinese Yuan, and responder receives 9 Chinese Yuan). Please see Text S2 for details about the envelopes and our experiment settings.

After taking the envelope, each participant, also one at a time, went into a separate cubicle beside the classroom to make a decision. In the sessions of experimenter condition, there was one experimenter in the cubicle who observed each participant’s decision-making behavior. The participant was not allowed to communicate with the experimenter. In the sessions of anonymous condition, there was no one in the cubicle. Two games were carried out in a given condition in each session. In the cubicle, (a) in the behavioral paradigm, the participant made a decision upon the “real” proposal according to the rules (informed IG or non-informed IG), if the participant accepted the proposal, he/she would take the money; otherwise, he/she would leave the money in the envelope; (b) in the questionnaire paradigm, the participant decided whether to accept or reject each of the nine possible proposals (ranging from 9∶1 to 1∶9) by marking (printing with a stamp) their decision on the corresponding place on the sheet. Participants did not get money when responding to the questionnaire paradigm, and their actual outcomes were determined only by whether they accepted or rejected the proposal (took the money or left it in the envelope).

Thereafter, the participant sealed the envelope, went back into the classroom, and dropped the envelope into a ballot box (a box with a narrow split on the top). The participant could then leave or go back to his/her seat in the classroom. Only after one participant finished all steps above could the next participant start the task. Participants who stayed in the class room were not allowed to communicate. With this procedure, in the non-informed IG under anonymous condition, no one (including the experimenter and the proposer) could know a given participant’s individual decision; therefore, each participant could trust that the decision-making was truly private. As we reasoned in the part of Introduction, responders’ rejection of unfair offers in the non-informed IG under anonymous condition could *not* be attributed to the responders’ deliberate cognitive processing of the consequence of their rejection behavior, e.g. reputational concerns, but could be driven by negative emotions.

According to the condition in each session and the envelope type taken by each participant, all 427 participants were assigned to the anonymous condition (*n* = 92 for informed-IG and 92 for non-informed-IG) or the experimenter condition (*n* = 127 for informed-IG and 116 for non-informed-IG). They all completed the decision sheet in the questionnaire paradigm. To elicit responders’ decision on unfair offers in the questionnaire paradigm, about 85% of the envelopes contained “real” proposals of 8∶2 (proposer received 8 Chinese Yuan, and responder received 2 Chinese Yuan. The decisions on 8∶2 proposals were also used as participants’ decisions on unfair offers in Yamagishi’s study (800, 200 Japanese Yen) [14]). To enhance the sense of reality, some envelopes in the box contained other proposals (7∶3, 6∶4, and 5∶5; about 5% each). As some participants took “real” proposals other than 8∶2, decisions on 8∶2 “real” proposals were obtained from 355 participants, including the anonymous condition (*n* = 78 in informed-IG and *n* = 83 in non-informed-IG), and the experimenter condition (98 in informed-IG and 96 in non-informed-IG). The results from these 355 participants were regarded as the responders’ decisions toward unfair offers in the behavioral paradigm.

## Results

In the behavioral paradigm, participants were led to believe that they were facing a single offer that had actually been proposed by a “real” proposer and that they should choose whether to accept or reject it. Participants’ decisions on 8∶2 proposals (proposer received 8 Chinese Yuan, and responder received 2 Chinese Yuan) were obtained as their decisions on unfair offers in each game in each condition.

**Figure 1:**
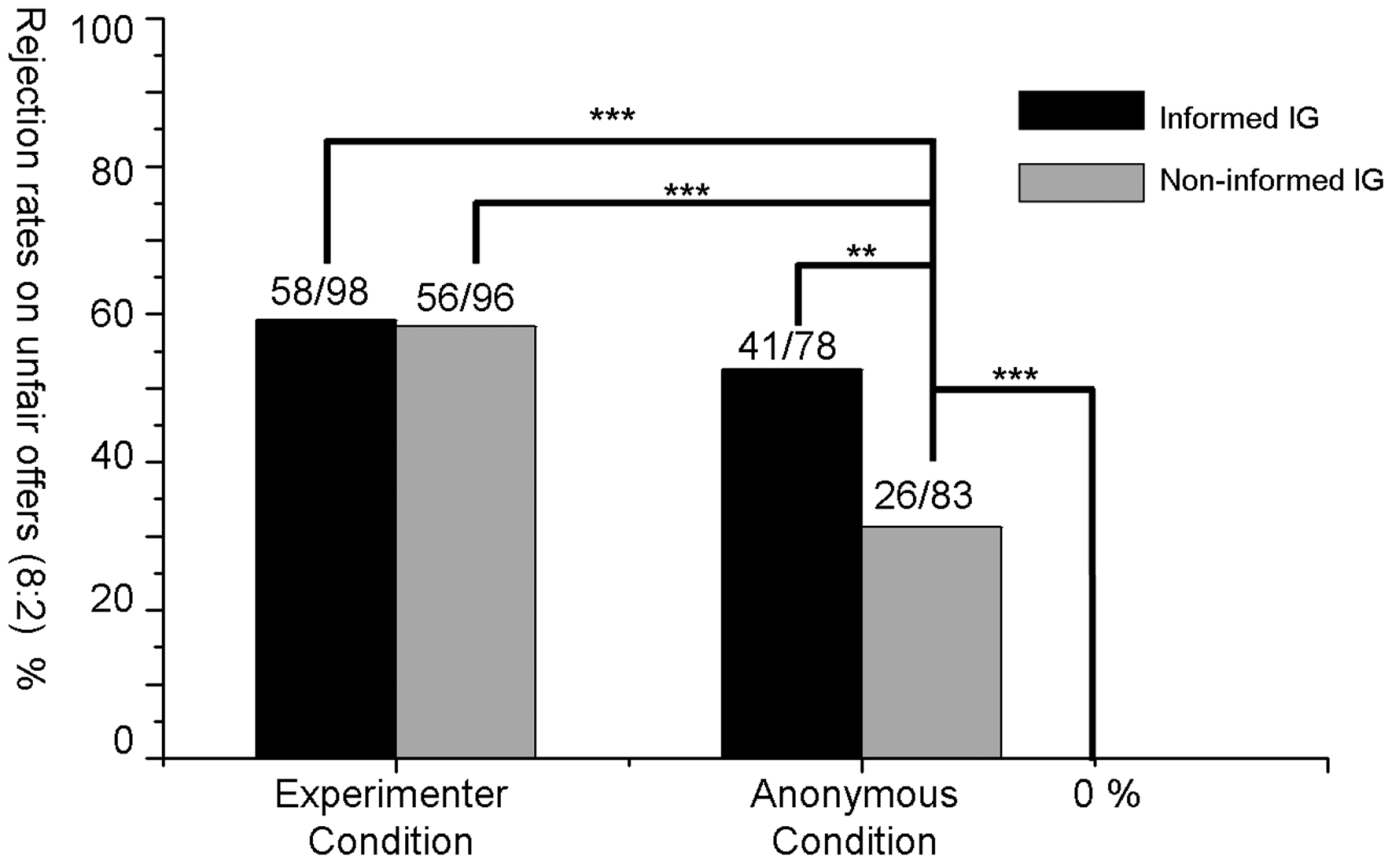
Rejection rates of unfair offers (8∶2) in the behavioral paradigm. It is clear that the rejection rates in the three public situations (informed-IG and non-informed-IG in the experimenter condition and the informed-IG in the anonymous condition) were significantly larger than the rejection rate in the private situation (non-informed-IG in the anonymous condition), and there was a substantial rejection rate in the private situation. Numbers on top of the bars (e.g., 58/98) represent the numbers of participants who rejected the offers (e.g., 58) and the total numbers of participants in that game in the condition (e.g., 98). *** Significantly differed at *p*<0.001 level, and ** at *p*<0.01 level in χ^2^ tests.

As shown in Figure 1, in the experimenter condition, the rejection rate for the 8∶2 offer in the informed-IG (59.2%) was not significantly different from that of the non-informed-IG (58.3%, χ^2^ (1)  = 0.014 *p = *1.000, ns.). However, in the anonymous condition, the rejection rate in the informed-IG (52.6%) was significantly higher than that in the non-informed-IG (31.3%, χ^2^ (1)  = 7.465 *p = *0.007), and the rejection rate in the non-informed-IG was significantly larger than zero (χ^2^ (1)  = 30.829 *p<*0.001).

The rejection rate in the non-informed-IG in the anonymous condition was also significantly lower than those in informed-IG and non-informed-IG in the experimenter condition (χ^2^ (1)  = 14.023 *p<*0.001, and χ^2^ (1)  = 13.080 *p<*0.001), and the rejection rate in the informed-IG in the anonymous condition was not significantly different from those in the informed-IG and non-informed-IG in the experimenter condition (*p*>0.4, ns.).

In the questionnaire paradigm, participants were asked to imagine they were facing a range of offers, and they had to decide whether to accept or reject each of the nine possible offers. The results, which were summarized in Table 1, replicated the findings in our behavioral paradigm. Toward unfair offers (8∶2 and 7∶3), participants in the non-informed-IG in the anonymous condition (35.9% and 35.9%, rejection rates for 8∶2 and 7∶3, respectively) showed significantly lower rejection rates than those in the informed IG in the anonymous condition (63.0% and 56.5%), informed IG (66.1% and 57.5%) and non-informed IG (61.2% and 53.4%) in the experimenter condition, and the rejection rates toward these unfair offers in the non-informed-IG in the anonymous condition were significantly larger than zero. Please see Text S3 for detailed results of rejection rates in questionnaire paradigm.

**Table 1:** Rejection rates in questionnaire paradigm.

Offers in		Rejection rates (%)			
Chinese Yuan		Experimenter condition		Anonymous condition	
Proposergot	Respondergot	Informed IGN = 127 *	Non-informed IG116	Informed IG92	Non-informed IG92
9	1	78.0	80.2	75.0	62.0
8	2	66.1	61.2	63.0	35.9 **
7	3	57.5	53.4	56.5	35.9 **
6	4	33.9	31.0	27.2	16.3
5	5	11.0	18.1	9.8	7.6
4	6	22.8	21.6	17.4	17.4
3	7	29.9	34.5	27.2	22.8
2	8	34.6	37.1	27.2	23.9
1	9	32.3	43.1	31.5	26.1

* Total numbers of participants in the corresponding game and condition.

** For those offers, participants’ rejection rates in the non-informed IG in anonymous condition were (a) significantly lower than those in informed IG in the anonymous condition, and the informed IG and non-informed IG in the experimenter condition (*p*≤0.012, in χ^2^ tests) and (b) significantly larger than zero (*p*<0.001, in χ^2^ tests). See Supporting Text S3 for details.

## Discussion

We obtained people’s rejection rates of unfair offers in informed-IG and non-informed-IG in experimenter and anonymous conditions. Results were consistent across behavioral and questionnaire paradigms. In the experimenter condition, responders’ rejection rates were not significantly different between informed-IG and non-informed-IG, which is similar to the pattern in Yamagishi’s results. In the anonymous condition, however, the rejection rate in non-informed-IG was significantly larger than zero and lower than that in informed-IG.

According to our hypothesis, we isolated the effects of reputational concerns and negative emotions on rejection of unfair offers. In the non-informed-IG under the anonymous condition, since responders believed that no one would know their individual choices, their rejection could not be attributed to the concerns of protecting personal reputation, but was driven by negative emotions. The substantial rejection rate in this situation, together with Yamagishi’s findings, provided more complete behavioral evidence suggesting that rejection of unfair offers can be driven by negative emotions.

### Biological Basis of Negative Emotions on Rejection of Unfair Offers

Some recent studies have explored the biological basis of social decision-making [1], [2], [4], [6], [19], [20], [21], [22], [23], [24], [25]. To our knowledge, no neural imaging study with true anonymous settings directly investigates the neural correlates of the rejection of unfair offers that is driven by negative emotions2. However, some studies found that in response to unfair offers, people usually exhibit negative emotions such as anger and outrage and show concomitant physiological and neural responses [26]. For example, people confronted with unfair behaviors showed increased activity in the anterior insula, a brain area associated with negative emotions, and the strength of the activation was positively correlated with rejection rates [2], [4]. These findings suggested that the desire to alleviate unpleasant feelings evoked by unfair acts may be one of the primary motivations for the rejection of unfair offers [2]. Therefore, from a biological perspective, it could be suggested that rejection of unfair offers could be driven by negative emotions, and that brain activation and physiological responses related to affect are part of underlying biological processes.

### Effects of Cognition on Rejection

In the present study, responders’ rejection rates in public situations (informed-IG in the anonymous condition, and informed-IG and non-informed-IG in the experimenter condition) were significantly larger than rejection rates in the private situation (non-informed-IG in the anonymous condition). This increase may be due to the effect of responders’ cognitive processing of their behavioral consequences. Cognitive functions that specifically deal with the social environment can acquire and process information from the environment. Thus, a small change in the environment could make a big difference in behavior [10]. For example, people rejected more unfair offers from human proposers than those randomly provided by a computer [27], [28], suggesting a top-down cognitive influence on the processing of unfair offers. In the public situations in our present study, responders could utilize cognitive abilities to understand that their decisions would be known by others and conclude that rejection has positive consequences, such as protecting personal reputation, thus reject unfair offers. As a result, in public situations, the rejection rates were significantly increased.

### Functions of Emotion and Cognition

It has been suggested that when people make social decisions in a rich and interactive environment, both affective factors (such as negative emotions) and cognition are functioning [2], [6]. Together with Yamgishi’s and our present findings, it could be inferred that pro-social or altruistic behaviors, such as maintaining fairness and cooperation, could be driven by affective factors without deliberate reasoning of the behavioral consequence. This raises the possibility that people could exhibit these behaviors with limited cognitive resources, which can be seen as a type of protection of these behaviors that guards the *stability* of social decision-making. This notion is in line with Yamagishi’s suggestion that emotion can be “as a commitment device” to ensure the consistence of people’s behavior on unfair offers [11]. On the other hand, cognitive functions that allow people to fully understand and evaluate the consequences of their behavior may lead to changes in decisions, e.g., by motivating people to be less selfish and more strategic with consideration of social factors such as reciprocity and equity [10]. Therefore, it can be suggested that cognition ensures the *efficiency* of social decision-making behaviors in complicated and flexible situations.

In conclusion, we found that people’s rejection rates of unfair offers were significantly larger than zero in a private situation, which provided further evidence suggesting that rejection of unfair offers can be driven by negative emotions. The higher rejection rates in public situations suggested that deliberate cognitive processing of the consequences of the behavior, such as reputational concerns, could increase the rejection rate, which may benefit social cooperation.

### Limitations and Future Work

Behavioral, brain imaging and neuropsychological studies in future are needed to further clarify the behavioral characteristics, biological basis, and individual differences in rejection of unfair offers, so as to explore the nature of the behaviors in social decision-making under given situations. Especially, to assure anonymity, the present study did not obtain any debriefing data (such as survey evidence) to quantitatively test whether participants indeed experienced negative emotions when they were confronted with unfair offers. Future studies with more sophisticated design which can get participants’ emotion reactions under anonymity are asking for. Moreover, people usually exhibit more altruism behaviors as the “social distance” decrease [29], [30], which may be one of the possible interpretations for the larger rejection rates in our pubic situations. However, as there is generally only a rather limited range for perceived social distance in economics experiments maintaining anonymity among the participants [30], it was not easy to clarify the effect of social distance on rejection rates based on our current results, which is an important question needed to study in the future.

**Note 2.** In a recent review [6], researchers mentioned that “Both anonymous and nonanonymous versions of the above games (including UG) have been studied with neuroimaging” (page 26). The term “anonymous” used in that review was specific to the anonymity between partners in the games, rather than each participant’s anonymity as in our present study.

## Supporting Information

Text S1 **Information about each testing session.**
(PDF)

Text S2 **Information about the envelopes and our experiment settings.**
(PDF)

Text S3 **Statistic results of rejection rates in questionnaire paradigm.**
(PDF)
