# Supplementary material for: Rejection of Unfair Offers Can Be Driven by Negative Emotions, Evidence from Modified Ultimatum Games with Anonymity
Source: PLoS One. 2012 Jun 28;7(6):e39619. doi: 10.1371/journal.pone.0039619 (PMC3386255; doi:10.1371/journal.pone.0039619)
Supplement: Text S1 — Information about each testing session. (PDF) [file pone.0039619.s001.pdf]

Supporting Text S1, Information about each testing session

As described in Method section, our data were obtained in several testing sessions. In each testing session, two games (Informed IG and Non-informed IG) were carried out in a given condition (Experimenter or Anonymous condition). Please see details in Table SI1.1 and SI1.2.

Table S1.1 Data of each testing session in Experimenter Condition

Session	All of participants	Informed IG No. of participants	Informed IG * 8:2 proposal	Informed IG ** Reject 8:2 proposal	Non-informed IG No. of participants	Non-informed IG * 8:2 proposal	Non-informed IG ** Reject 8:2 proposal
1	76	41	33	17	35	27	14
2	70	34	25	16	36	35	21
3	65	42	35	24	23	15	9
4	32***	10	5	1	22	19	12
Total	243	127	98	58	116	96	56

Table S1.2 Data of each testing session in Anonymous Condition

Session	All of participants	Informed IG No. of participants	Informed IG * 8:2 proposal	Informed IG ** Reject 8:2 proposal	Non-informed IG No. of participants	Non-informed IG * 8:2 proposal	Non-informed IG ** Reject 8:2 proposal
1	66	32	29	16	34	29	11
2	15 ***	11	8	4	4	4	1
3	21 ***	11	9	7	10	7	2
4	11 ***	5	5	2	6	6	2
5	12 ***	7	7	2	5	5	2
6	59	26	20	10	33	32	8
Total	184	**92**	**78**	**41**	**92**	**83**	**26**

*** 8:2 proposal**: The number of participants who ***confronted with*** 8:2 proposal within all participants in behavioral paradigm in the session.

**** Reject 8:2 proposal**: The number of participants who ***reject*** 8:2 proposal within participants who confronted with 8:2 proposal in behavioral paradigm in the session.

******* When the data with relatively smaller sample size (the sessions with less than 50 participants, including session 4 in experimenter condition and session 2, 3, 4, 5 in anonymous condition**)** were excluded, we got similar results as the data from all testing sessions, as shown in SI 1.3 and SI 1.4.

For data summed up from (a) all testing sessions (shown in SI 1.3) and (b) only from testing sessions with relatively larger sample size (testing sessions with more than 50 participants, SI 1.4), $\chi 2$ –tests were performed between the rejection-rate (for 8:2 proposal in behavioral paradigm) pairs in (1) informed IG in experimenter condition, (2) non-informed IG in the experimenter

condition, (3) informed IG in the anonymous condition, (4) non-informed IG in the anonymous condition, and between each rejection rate and zero. Significantly difference at $p < 0.05$ level were shown in **Bold**.

Table S1.3 Statistic results of data summed up from all testing sessions.

	**Experimenter condition**				**Anonymous condition**			
	Informed IG		Non-informed IG		Informed IG		Non-informed IG	
Total number of participants	98		96		78		83	
No. and % of participants	58		56		41		26	
who reject the offer	59.2%		58.3%		52.6%		31.3%	
χ^2 -test	χ^2	*p*	χ^2	*p*	χ^2	*p*	χ^2	*p*
**Experimenter condition**								
Informed IG, 59.2%	-	-	0.014	1.000	0.773	0.445	**14.023**	**<0.001**
Non-informed IG, 58.3%			-	-	0.581	0.539	**13.080**	**<0.001**
**Anonymous condition**								
Informed IG, 52.6%					-	-	**7.465**	**0.007**
Non-informed IG, 31.3%							-	-
**Zero**	**82.38**	**<.001**	**79.06**	**<.001**	**55.62**	**<.001**	**30.83**	**<.001**

Table S1.4 Statistic results of data summed up from testing sessions with relatively larger sample size, i.e., session 1, 2, 3 in Experimenter Condition and session 1, 6 in Anonymous Condition.

	**Experimenter condition**				**Anonymous condition**			
	Informed IG		Non-informed IG		Informed IG		Non-informed IG	
Total number of participants	93		77		49		61	
No. and % of participants	57		44		26		19	
who reject the offer	61.3%		57.1%		53.1%		31.1%	
χ^2 -test	χ^2	*p*	χ^2	*p*	χ^2	*p*	χ^2	*p*
**Experimenter condition**								
Informed IG, 61.3%	-	-	0.300	0.639	0.895	0.374	**13.390**	**<0.001**
Non-informed IG, 57.1%			-	-	0.202	0.715	**9.270**	**0.003**
**Anonymous condition**								
Informed IG, 53.1%					-	-	**5.398**	**0.031**
Non-informed IG, 31.1%							-	-
**Zero**	**82.19**	**<.001**	**61.60**	**<.001**	**38.39**	**<.001**	**22.50**	**<.001**

The patterns of statistic results were similar in SI 1.3 and SI 1.4, where participants' rejection rates in the non-informed IG in anonymous condition were (1) significantly lower than those in informed IG in the anonymous condition, and the informed IG and non-informed IG in the experimenter condition and (2) significantly larger than zero.
