# Supplementary material for: Rejection of Unfair Offers Can Be Driven by Negative Emotions, Evidence from Modified Ultimatum Games with Anonymity
Source: PLoS One. 2012 Jun 28;7(6):e39619. doi: 10.1371/journal.pone.0039619 (PMC3386255; doi:10.1371/journal.pone.0039619)

Supporting Text S2, Information about the envelopes and our experiment settings

As described in Method section, each envelope contained (1) the “rule”, which explained whether the participant was playing the informed IG or the non-informed IG; (2) the “real” proposal by a “proposer”: a number-stamp printed on the paper represented the money a proposer offered; (3) the money offered to the responder according to the proposal; and (4) a decision sheet with all nine possible proposals from 9:1 (proposer receives 9 Chinese Yuan, and responder receives 1 Chinese Yuan) to 1:9 (proposer receives 1 Chinese Yuan, responder receives 9 Chinese Yuan).

The “rule”, “real” proposal and decision sheet were printed on a sheet of paper in Chinese and sealed in the envelope (as shown in SI 2.1, SI 2.2 SI 2.3). The “rules” were different between informed IG and non-informed IG, and the “rules” of the two games were same between anonymous condition and experimenter condition.

Table S2.1 The “rule” and the “real” proposal for informed IG

INSTRUCTION

No. **123***

***Rule*：**

Whether the responder accepts or rejects the offer, the proposer (1) will get the money according to the proposal, and (2) will be informed of the responder’s choice (accept or reject).

If you are the proposer, please memorize the “**No.**” in the right-up corner and finish the part of “**Proposal**” with the number-stamp. We will connect you via the No. and carry out the “Rule” based on the responder’s choice.

If you are the responder, please make a decision on the “**Proposal**”: take the money (i.e., accept the proposal) or leave the money in the envelope (i.e., reject the proposal).

Proposal (filled by proposer):

Proposer get: **8**** Yuan; Responder get: **2** Yuan

If you are the responder, please also **finish the decision sheet** in the other side of this paper with the stamp ***.

***** the No. was a 3-digits number printed with number-stamps, and the No. were different across each paper.

****** the “real” proposal by a “proposer”: printed with a number-stamp represented the money a proposer offered.

******* to further ensure the responder’s anonymity, the stamp was used instead of hand-writing.

Table S2.2 The "rule" and the "real" proposal for Non-informed IG

INSTRUCTION

No. **123**

Rule:

Whether the responder accepts or rejects the offer, the proposer (1) will get the money according to the proposal, and (2) will **NOT** be informed of the responder's choice (accept or reject).

If you are the proposer, please memorize the "**No.**" in the right-up corner and finish the part of "**Proposal**" with the number-stamp. We will connect you via the No. and carry out the "Rule" based on the responder's choice.

If you are the responder, please make a decision on the "**Proposal**": take the money (i.e., accept the proposal) or leave the money in the envelope (i.e., reject the proposal).

Proposal (filled by proposer):

Proposer get: **8** Yuan; Responder get: **2** Yuan

If you are the responder, please also **finish the decision sheet** in the other side of this paper with the stamp.

Table S2.3 The decision sheet

If you are the responder, please finish this sheet based on the “**rule**”: make a choice for each of nine possible proposals.

As a responder, if you can accept the proposal, please mark the corresponding place in the right column with the stamp, if you **can not** accept, please **do not** leave any mark.

**Proposer Gets(Yuan)**	**You Get(Yuan)**	**OK?**	**Proposer Gets(Yuan)**	**You Get(Yuan)**	**OK?**
**9**	**1**		**1**	**9**	
**8**	**2**		**2**	**8**	
**7**	**3**		**3**	**7**	
**6**	**4**		**4**	**6**	
**5**	**5**		**--**	**--**	**--**

Figure S2.1 our experiment settings

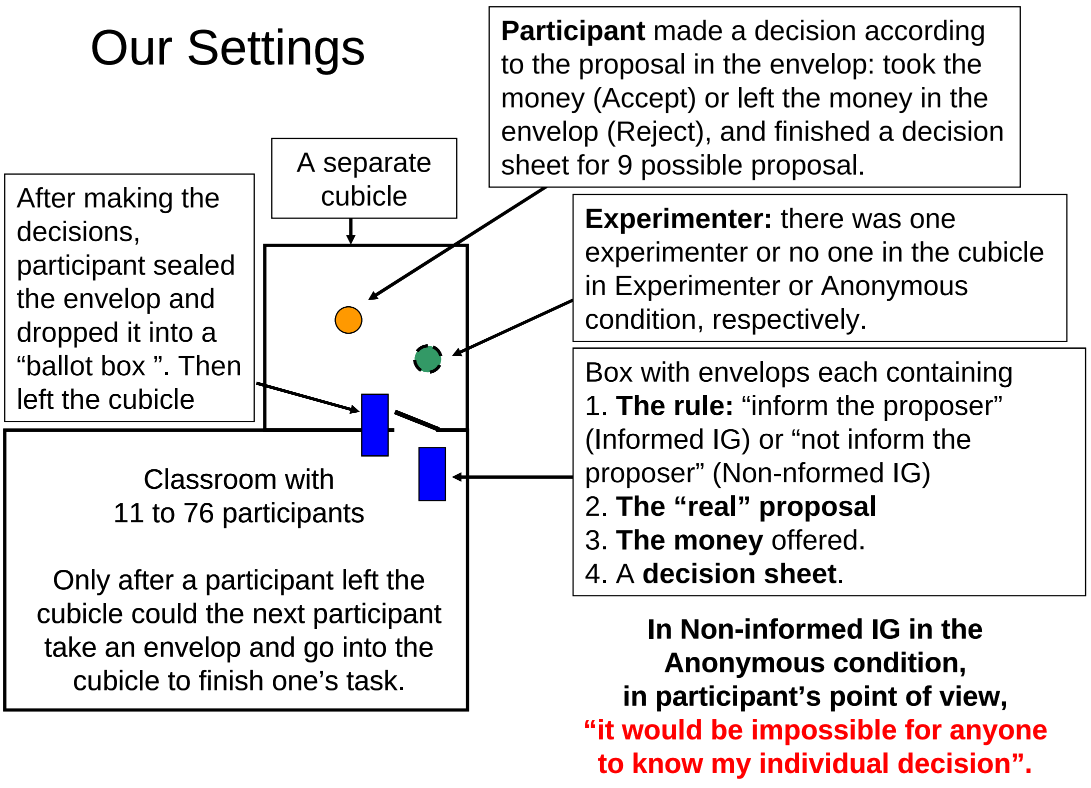

Supplement: Text S2 — Information about the envelopes and our experiment settings. (PDF) [file pone.0039619.s002.pdf]
