# Supplementary material for: Rejection of Unfair Offers Can Be Driven by Negative Emotions, Evidence from Modified Ultimatum Games with Anonymity
Source: PLoS One. 2012 Jun 28;7(6):e39619. doi: 10.1371/journal.pone.0039619 (PMC3386255; doi:10.1371/journal.pone.0039619)
Supplement: Text S3 — Statistic results of rejection rates in questionnaire paradigm. (PDF) [file pone.0039619.s003.pdf]

Supporting Text S3, Statistic results of rejection rates in questionnaire paradigm

For each of 9 proposals in questionnaire paradigm, χ^2 –tests were performed between the rejection-rate pairs in (1) informed IG in experimenter condition, (2) non-informed IG in the experimenter condition, (3) informed IG in the anonymous condition, (4) non-informed IG in the anonymous condition, and between each rejection rate and zero. As shown in SI 3.1 to SI 3.10 (Significantly difference at $p < 0.05$ level were shown in **Bold)**.

For 9:1, 8:2, 7:3 proposals, the rejection rate in non-informed IG in anonymous condition (a) were significantly (with one exception at 0.080 level) lower than those in informed IG in the anonymous condition, and the informed IG and non-informed IG in the experimenter condition, and (b) significantly larger than zero.

Figure S3.1, Rejection rates in questionnaire paradigm

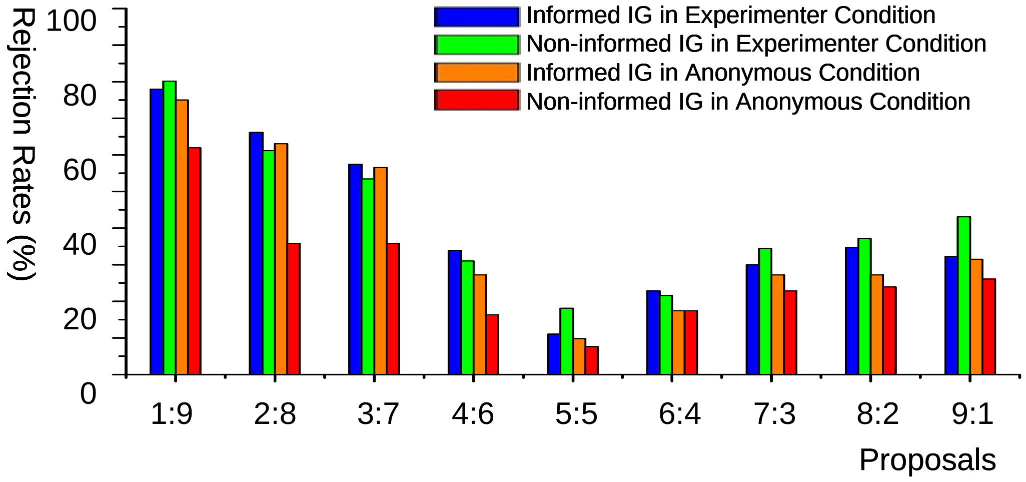

Table S3.1, 9:1 proposal

Proposer, **9 yuan**	**Experimenter condition**				**Anonymous condition**			
Responder, **1 yuan**	Informed IG		Non-informed IG		Informed IG		Non-informed IG	
Total number of participants	127		116		92		92	
No. and % of participants	99		93		69		57	
who reject the offer	78.0%		80.2%		75.0%		62.0%	
**χ^2 -test**	χ^2	*p*	χ^2	*p*	χ^2	*p*	χ^2	*p*
**Experimenter condition**								
Informed IG, 78.0%	-	-	0.180	0.753	0.260	0.630	**6.662**	**0.015**
Non-informed IG, 80.2%			-	-	0.797	0.403	**8.466**	**0.004**
**Anonymous condition**								
Informed IG, 75.0%					-	-	3.626	0.080
Non-informed IG, 62.0%							-	-
**Zero**	**162.2**	**<.001**	**155.2**	**<.001**	**110.4**	**<.001**	**82.58**	**<.001**

Table S3.2, 8:2 proposal

Proposer, **8 yuan**	**Experimenter condition**				**Anonymous condition**			
Responder, **2 yuan**	Informed IG		Non-informed IG		Informed IG		Non-informed IG	
Total number of participants	127		116		92		92	
No. and % of participants	84		71		58		33	
who reject the offer	66.1%		61.2%		63.0%		35.9%	
**χ^2 -test**	χ^2	*p*	χ^2	*p*	χ^2	*p*	χ^2	*p*
**Experimenter condition**								
Informed IG, 66.1%	-	-	0.639	0.504	0.225	0.669	**19.649**	**< 0.001**
Non-informed IG, 61.2%			-	-	0.073	0.886	**13.175**	**< 0.001**
**Anonymous condition**								
Informed IG, 63.0%					-	-	**13.589**	**< 0.001**
Non-informed IG, 35.9%							-	-
**Zero**	**125.5**	**<.001**	**102.3**	**<.001**	**84.70**	**<.001**	**40.21**	**<.001**

Table S3.3, 7:3 proposal

Proposer, **7 yuan**	**Experimenter condition**				**Anonymous condition**			
Responder, **3 yuan**	Informed IG		Non-informed IG		Informed IG		Non-informed IG	
Total number of participants	127		116		92		92	
No. and % of participants	73		62		52		33	
who reject the offer	57.5%		53.4%		56.5%		35.9%	
χ^2 **-test**	χ^2	*p*	χ^2	*p*	χ^2	*p*	χ^2	*p*
**Experimenter condition**								
Informed IG, 57.5%	-	-	0.399	0.605	0.020	0.891	**9.977**	**0.002**
Non-informed IG, 53.4%			-	-	0.196	0.676	**6.390**	**0.012**
**Anonymous condition**								
Informed IG, 56.5%					-	-	**7.894**	**0.008**
Non-informed IG, 35.9%							-	-
**Zero**	**102.4**	**<.001**	**84.61**	**<.001**	**72.48**	**<.001**	**40.21**	**<.001**

Table S3.4, 6:4 proposal

Proposer, **6 yuan**	**Experimenter condition**				**Anonymous condition**			
Responder, **4 yuan**	Informed IG		Non-informed IG		Informed IG		Non-informed IG	
Total number of participants	127		116		92		92	
No. and % of participants	43		36		25		15	
who reject the offer	33.9%		31.0%		27.2%		16.3%	
χ^2 **-test**	χ^2	*p*	χ^2	*p*	χ^2	*p*	χ^2	*p*
**Experimenter condition**								
Informed IG, 33.9%	-	-	0.220	0.682	1.113	0.305	**8.444**	**0.005**
Non-informed IG, 31.0%			-	-	0.369	0.646	**6.015**	**0.015**
**Anonymous condition**								
Informed IG, 27.2%					-	-	3.194	0.107
Non-informed IG, 16.3%							-	-
**Zero**	**51.76**	**<.001**	**42.61**	**<.001**	**28.93**	**<.001**	**16.33**	**<.001**

Table S3.5, 5:5 proposal

Proposer, **5 yuan**	**Experimenter condition**				**Anonymous condition**			
Responder, **5 yuan**	Informed IG		Non-informed IG		Informed IG		Non-informed IG	
Total number of participants	127		116		92		92	
No. and % of participants	14		21		9		7	
who reject the offer	11.0%		18.1%		9.8%		7.6%	
**χ^2 -test**	χ^2	p	χ^2	p	χ^2	p	χ^2	p
**Experimenter condition**								
Informed IG, 11.0%	-	-	2.465	0.144	0.087	0.826	0.718	0.489
Non-informed IG, 18.1%			-	-	2.878	0.112	**4.851**	**0.039**
**Anonymous condition**								
Informed IG, 9.8%					-	-	0.274	0.798
Non-informed IG, 7.6%							-	-
**Zero**	**14.82**	**<.001**	**23.09**	**<.001**	**9.463**	**0.013**	**7.277**	**0.014**

Table S3.6, 4:6 proposal

Proposer, **4 yuan**	**Experimenter condition**				**Anonymous condition**			
Responder, **6 yuan**	Informed IG		Non-informed IG		Informed IG		Non-informed IG	
Total number of participants	127		116		92		92	
No. and % of participants	29		25		16		16	
who reject the offer	22.8%		21.6%		17.4%		17.4%	
**χ^2 -test**	χ^2	p	χ^2	p	χ^2	p	χ^2	p
**Experimenter condition**								
Informed IG, 22.8%	-	-	0.058	0.878	0.968	0.398	0.968	0.398
Non-informed IG, 21.6%			-	-	0.561	0.487	0.561	0.487
**Anonymous condition**								
Informed IG, 17.4%					-	-	0.000	1.000
Non-informed IG, 17.4%							-	-
**Zero**	**32.74**	**<.001**	**28.02**	**<.001**	**17.52**	**<.001**	**17.52**	**<.001**

Table S3.7, 3:7 proposal

Proposer, **3 yuan**	**Experimenter condition**				**Anonymous condition**			
Responder, **7 yuan**	Informed IG		Non-informed IG		Informed IG		Non-informed IG	
Total number of participants	127		116		92		92	
No. and % of participants	38		40		25		21	
who reject the offer	29.9%		34.5%		27.2%		22.8%	
**χ^2 -test**	χ^2	*p*	χ^2	*p*	χ^2	*p*	χ^2	*p*
**Experimenter condition**								
Informed IG, 29.9%	-	-	0.579	0.493	0.197	0.763	1.365	0.281
Non-informed IG, 34.5%			-	-	1.276	0.293	3.364	0.091
**Anonymous condition**								
Informed IG, 27.2%					-	-	0.464	0.610
Non-informed IG, 22.8%							-	-
**Zero**	**44.69**	**<.001**	**48.33**	**<.001**	**28.93**	**<.001**	**23.71**	**<.001**

Table S3.8, 2:8 proposal

Proposer, **2 yuan**	**Experimenter condition**				**Anonymous condition**			
Responder, **8 yuan**	Informed IG		Non-informed IG		Informed IG		Non-informed IG	
Total number of participants	127		116		92		92	
No. and % of participants	44		43		25		22	
who reject the offer	34.6%		37.1%		27.2%		23.9%	
**χ^2 -test**	χ^2	*p*	χ^2	*p*	χ^2	*p*	χ^2	*p*
**Experimenter condition**								
Informed IG, 34.6%	-	-	0.155	0.789	1.380	0.302	2.919	0.102
Non-informed IG, 37.1%			-	-	2.283	0.140	4.133	0.050
**Anonymous condition**								
Informed IG, 27.2%					-	-	0.257	0.736
Non-informed IG, 23.9%							-	-
**Zero**	**53.22**	**<.001**	**52.78**	**<.001**	**28.93**	**<.001**	**24.99**	**<.001**

Table S3.9, 1:9 proposal

Proposer, **1 yuan**	**Experimenter condition**				**Anonymous condition**			
Responder, **9 yuan**	Informed IG		Non-informed IG		Informed IG		Non-informed IG	
Total number of participants	127		116		92		92	
No. and % of participants	41		50		29		24	
who reject the offer	32.3%		43.1%		31.5%		26.1%	
**χ^2 -test**	χ^2	p	χ^2	p	χ^2	p	χ^2	p
**Experimenter condition**								
Informed IG, 32.3%	-	-	3.030	0.086	0.014	1.000	0.982	0.370
Non-informed IG, 43.1%			-	-	2.922	0.113	6.842	0.013
**Anonymous condition**								
Informed IG, 31.5%					-	-	0.663	0.515
Non-informed IG, 26.1%							-	-
**Zero**	**48.89**	**<.001**	**63.74**	**<.001**	**34.43**	**<.001**	**27.60**	**<.001**
